# Peripheral immune profiles associated with an operational atopy-related phenotype in young infants with pertussis: a single-center retrospective study

**DOI:** 10.3389/fped.2026.1987917

**Published:** 2026-10-29

**Authors:** Lingyun Zhang, Yanhong Ren, Jun Wang

**Affiliations:** 1 Department of Emergency Medicine, Children's Hospital Affiliated to Zhengzhou University, Henan Children's Hospital, Zhengzhou Children's Hospital, Zhengzhou, China; 2 Henan Province Engineering Research Center of Diagnosis and Treatment of Pediatric Infection and Critical Care, Zhengzhou, China

**Keywords:** interferon-gamma, interleukin-6, length of stay, operational phenotype, pertussis, T-lymphocyte subsets, young infants

## Abstract

**Background:**

Host immune responses to pertussis may vary among young infants, but the relevance of concurrently recorded atopy-related features is uncertain.

**Methods:**

This single-center retrospective study included 120 infants aged 1–3 months with pertussis (75 PCR-confirmed and 45 clinically diagnosed with documented epidemiological contact) and 50 pertussis-PCR-negative pneumonia disease controls. A prespecified operational atopy-related phenotype (OAP) required total IgE >15 IU/mL plus eosinophilia, eczema, or parental allergic history. Primary pertussis-only models compared 45 OAP + with 75 OAP− infants after adjustment for chronological age, sex, PCR status, cough duration before sampling, and admission year; false-discovery-rate adjustment was applied within lymphocyte and cytokine outcome families.

**Results:**

OAP + status was associated with a higher CD4 + percentage (adjusted mean difference 5.25 percentage points, 95% CI 2.92–7.57), lower CD8 + percentage (−2.36, −4.38 to −0.34), and higher CD4/CD8 ratio (0.44, 0.21–0.68). OAP + infants had lower IFN-*γ* (adjusted geometric mean ratio 0.72, 0.58–0.90), higher IL-6 (2.07, 1.65–2.59), lower IL-2 (0.70, 0.52–0.94), and a lower IFN-*γ*/IL-6 ratio (0.35, 0.25–0.48); all corresponding q values were <0.05. Core findings persisted under a stricter phenotype definition and in PCR-confirmed cases. In an exploratory negative-binomial model, OAP + status was associated with longer hospitalization (adjusted incidence rate ratio 1.33, 1.14–1.55), whereas the continuous cytokine ratio was not (0.98 per doubling, 0.92–1.03).

**Conclusion:**

A locally defined atopy-related phenotype was associated with selected peripheral immune differences and longer hospital stay in young infants with pertussis. These hypothesis-generating associations do not establish an atopy diagnosis, a pertussis-specific mechanism, or prognostic utility.

## 1 Introduction

Pertussis remains an important cause of hospitalization and death in young infants worldwide (1–3). China reported 476,690 pertussis cases and 30 deaths in 2024, compared with 41,124 cases and 5 deaths in 2023, marking a substantial national resurgence (4, 5). During most of the study period, the national schedule used acellular diphtheria-tetanus-pertussis vaccine at 3, 4, 5, and 18 months; from 1 January 2025, the schedule changed to 2, 4, 6, and 18 months with an additional dose at 6 years (6). Maternal pertussis vaccination was not part of this national routine schedule. Consequently, infants aged 1–3 months may be unvaccinated or may have received only an initial dose, although individual infant and maternal vaccination histories were unavailable in this retrospective dataset.

Young infants can show marked heterogeneity in inflammatory and lymphocyte responses during pertussis (7, 8). Natural *Bordetella pertussis* infection at 2 months of age has been associated with a predominantly type 1 T-cell response characterized by IFN-*γ* production (9). Conversely, early-onset atopic dermatitis is characterized by type 2-centered inflammation with additional type 17 and type 22 activity and relatively limited type 1 upregulation (10). Eczema, eosinophilia, elevated total IgE, and parental allergic disease are therefore plausible markers of an atopy-related host background, but none is independently diagnostic of atopy in infants during acute infection.

Whether such routinely recorded features identify immune heterogeneity among infants with pertussis has received little clinical study. A disease-control group with pneumonia can provide context for whether observed values are unique to one hospitalized infection cohort, but a phenotype-negative pneumonia group cannot test a pertussis-specific interaction. The clinically defensible question is therefore whether a prespecified, locally defined phenotype is associated with peripheral immune measurements within pertussis after accounting for available confounders.

We compared lymphocyte subsets and four serum cytokines between OAP + and OAP− infants with pertussis, using pneumonia controls for descriptive context. We prespecified two outcome families, adjusted the pertussis-only comparisons for available baseline covariates, and performed sensitivity analyses using stricter phenotype definitions and PCR-confirmed cases. Associations with hospital length of stay were exploratory.

## 2 Materials and methods

### 2.1 Study design and setting

This single-center retrospective comparative study used electronic medical records and laboratory information system data from Henan Children's Hospital, Zhengzhou, China. We screened admissions to the Departments of Infectious Diseases and Respiratory Medicine from January 2020 through December 2025. Chronological age, rather than corrected age, was used. Reporting followed the STROBE statement (11).

### 2.2 Pertussis cohort and case ascertainment

Eligible pertussis cases were 1–3 months of chronological age and had either a positive *Bordetella pertussis* PCR result or fulfilled the 2024 Chinese clinical diagnostic criteria with a documented epidemiological contact (1). All 120 included infants underwent an admission-day pharyngeal swab for pertussis PCR. The cohort included 75 PCR-confirmed cases and 45 PCR-negative clinical cases; every PCR-negative clinical case had documented contact with a person with suspected or confirmed pertussis.

Exclusion criteria were an inborn error of immunity; glucocorticoid or immunosuppressant use in the preceding 2 weeks; another clearly documented severe acute infection; a severe underlying disorder; or incomplete IgE, eosinophil, lymphocyte-subset, or cytokine data within 48 h. Of 150 pertussis-coded discharges, 14 were excluded after clinical review and 16 for incomplete biomarkers, leaving 120. The five infants excluded for severe concurrent infection presented with respiratory failure and were transferred immediately to the general pediatric ICU before baseline immune testing. Infants who deteriorated after immune sampling and received care in the infectious-disease or respiratory specialty ICU remained in the cohort; hospital days included both ward and specialty-ICU days. Only the first eligible hospitalization was retained, and no interdepartmental transfers occurred between the two recruiting departments.

### 2.3 Pneumonia disease controls

Pneumonia controls were hospitalized infants aged 1–3 months with a discharge diagnosis containing pneumonia and a negative pertussis PCR result. Among 1,539 candidates, 97 repeated admissions, 103 records meeting clinical exclusions, 587 with incomplete IgE, eosinophil, T-cell, or cytokine data within 48 h, and 364 meeting the operational phenotype were excluded, leaving 388 eligible phenotype-negative controls. We first set a target of 22 female and 28 male controls to mirror the pertussis cohort's sex distribution, assigned Excel-generated random numbers within each sex stratum, and selected 50 while approximately matching the pertussis age-frequency distribution. Fifty was chosen pragmatically to approximate the OAP + sample of 45, and selection occurred before group immune comparisons were examined. Controls were disease controls, not healthy reference participants.

For descriptive characterization of the pneumonia cohort, the treating physician's final clinical judgment classified each case as viral, bacterial, mixed, or unidentified/undetermined by integrating symptoms, C-reactive protein and blood-count results, imaging, and available pathogen testing. Pathogen testing was clinically directed rather than protocolized; these categories were not used for inferential comparisons.

### 2.4 Operational atopy-related phenotype

The operational phenotype was defined before T-cell and cytokine results were examined. OAP + required total serum IgE >15 IU/mL and at least one of: eosinophils >0.4 × 10⁹/L, documented infantile eczema, or allergic rhinitis, asthma, atopic dermatitis, or food allergy in either parent. The IgE and eosinophil thresholds were the upper reference limits used by the hospital laboratory for infants aged 1–3 months. OAP− denotes failure to meet the complete composite and must not be interpreted as absence of atopy. The construct was used for stratification only and is not a validated allergy diagnosis.

### 2.5 Laboratory measurements and data collection

Pertussis DNA was detected qualitatively by real-time PCR targeting IS481 (Daan Gene Co., Ltd., Guangzhou, China) on a Roche LightCycler 480 platform (Roche Diagnostics, Basel, Switzerland). Cycle-threshold values and bacterial load were unavailable. Although nasopharyngeal specimens are generally preferred for pertussis PCR (12), this study used pharyngeal swabs according to local clinical practice; reduced sensitivity is therefore possible.

In the two departments, total IgE, eosinophils, peripheral lymphocyte subsets, and available cytokines were used as a routine combined immunologic and inflammatory assessment in selected young infants with suspected pertussis or pneumonia; tests were clinically ordered and were not triggered by the study phenotype. T-cell subsets were measured by flow cytometry on a BD FACSCanto II; serum IFN-*γ*, IL-6, TNF-α, and IL-2 were quantified with the BD CBA Human Th1/Th2 Cytokine Kit II. Total IgE was measured by chemiluminescence on the IMMULITE 2000, and blood counts on a Sysmex XN-9000. Immune measurements used for this study were obtained within 48 h of admission. Cough duration was the number of days before admission or immune sampling. Reasons for incomplete testing in excluded infants were not systematically recorded.

Extracted variables were chronological age, sex, admission year, pertussis PCR and epidemiological contact, cough duration before sampling, hospital days, specialty-ICU care, total IgE, eosinophils, eczema, parental allergic history, peak WBC, lymphocyte percentages, and cytokines. Prematurity and gestational age, individual vaccination status, pre-admission macrolide treatment, detailed respiratory support, standardized severity measures, and uniform coinfection testing were unavailable.

### 2.6 Outcomes and statistical analysis

The primary exposure was OAP + status within the pertussis cohort. Two outcome families were prespecified: six lymphocyte outcomes (CD3+, CD4+, CD8+, CD4/CD8 ratio, CD19+, and NK-cell percentages) and five cytokine outcomes (IFN-*γ*, IL-6, TNF-α, IL-2, and the IFN-*γ*/IL-6 ratio). Three-group comparisons with pneumonia controls were secondary and descriptive: normally distributed measurements were compared by one-way analysis of variance, skewed measurements by Kruskal–Wallis tests, and categorical variables by chi-square or Fisher exact tests. Significant overall tests were followed by Bonferroni-adjusted pairwise comparisons. Variables used to define the phenotype were presented without inferential *P* values.

Primary pertussis-only associations were estimated with linear regression using HC3 robust standard errors. Lymphocyte outcomes were modeled on their original scale and reported as adjusted mean differences. Cytokines and the ratio were natural-log transformed and exponentiated to adjusted geometric mean ratios. Models included chronological age, sex, PCR status, cough duration before sampling, and centered admission year. Benjamini–Hochberg false-discovery-rate correction was applied separately within the lymphocyte and cytokine families. Variance inflation factors were examined.

Sensitivity analyses repeated the primary models among PCR-confirmed cases and under a stricter phenotype requiring IgE >15 IU/mL plus at least two of eosinophilia, eczema, and parental allergic history. Component-based definitions were also examined. Available characteristics of the 16 biomarker-incomplete infants were compared with included cases using Mann–Whitney U or Fisher exact tests. Spearman correlations were exploratory, were not adjusted for multiplicity, and were interpreted by direction and uncertainty rather than qualitative strength labels.

Hospital days were analyzed continuously. Because a Poisson model showed overdispersion, a negative-binomial model included OAP status and log2-transformed IFN-*γ*/IL-6 ratio together with the same covariates. Incidence rate ratios (IRRs) and 95% CIs are reported. Gamma regression with a log link and the previous median-dichotomized logistic model were sensitivity analyses. Peak WBC was not included because it was a post-admission variable. No *a priori* sample-size calculation was performed: all eligible complete pertussis cases over the 6-year period were included, and the control count was pragmatic. No missing-value imputation was used. Tests were two-sided with *P* < 0.05; adjusted q < 0.05 defined statistical significance for primary outcome families. Analyses used SPSS 26.0 and independently scripted verification in Python 3.13 with SciPy and statsmodels.

### 2.7 Ethics

The study was conducted in accordance with the Declaration of Helsinki and approved by the Ethics Committee of Henan Children's Hospital (2025-KY-0140-002). The requirement for informed consent was waived because the study used retrospective, de-identified clinical data.

## 3 Results

### 3.1 Participant selection and case ascertainment

Demographic and clinical characteristics of the study groups are summarized in Table 1. The analysis included 120 infants with pertussis (OAP+, *n* = 45; OAP−, *n* = 75) and 50 phenotype-negative pneumonia controls (Figure 1). Among pertussis cases, 75 were PCR-confirmed (26 OAP + and 49 OAP−) and 45 were clinically diagnosed with documented contact (19 OAP + and 26 OAP−); PCR confirmation did not differ detectably by phenotype (Fisher exact *P* = 0.440). Eleven included infants received specialty-ICU care after immune sampling (6/45 OAP + and 5/75 OAP−; *P* = 0.327). In the pneumonia controls, the treating physician's final classifications were viral in 11 (22%), bacterial in 19 (38%), mixed in 13 (26%), and unidentified/undetermined in 7 (14%).

**Table 1:** Demographic and clinical characteristics of the study groups.

Characteristic	OAP + (*n* = 45)	OAP− (*n* = 75)	PC (*n* = 50)	Statistic	*P* value
Age, days	57 (48–66)	63 (48–72)	58 (51–78)	H = 2.564	0.277
Male sex, *n* (%)	26 (57.8)	41 (54.7)	28 (56.0)	*χ*^2^ = 0.111	0.946
Total IgE, IU/mL	74.4 (50.7–118.4)	11.0 (8.0–19.6)	10.7 (7.9–14.2)	—	Descriptive
Eosinophils, × 10⁹/L	0.60 (0.44–0.73)	0.21 (0.16–0.32)	0.16 (0.11–0.23)	—	Descriptive
Eczema history, *n* (%)	32 (71.1)	4 (5.3)	0 (0.0)	—	Descriptive
Parental allergic history, *n* (%)	23 (51.1)	8 (10.7)	3 (6.0)	—	Descriptive
Length of stay, days	14 (11–18)	11 (8–13)	10 (8–11)	H = 31.023	<0.001
Cough duration before sampling, days	20 (16–23)	19 (14–24)	16 (12–19)	H = 11.649	0.003
Peak WBC, × 10⁹/L	31.3 (19.2–39.5)	23.7 (15.4–33.8)	14.5 (9.9–18.6)	H = 40.651	<0.001
PCR-confirmed pertussis, *n* (%)	26 (57.8)	49 (65.3)	0 (0), by design	—	—
Clinical pertussis diagnosis with contact, *n* (%)	19 (42.2)	26 (34.7)	—	—	—
Specialty ICU care, *n* (%)	6 (13.3)	5 (6.7)	—	Fisher exact	0.327

Continuous variables are median (interquartile range); categorical variables are *n* (%). Overall *P* values are from Kruskal–Wallis, chi-square, or Fisher exact tests. IgE, eosinophils, eczema, and parental allergic history are components of the operational phenotype and are shown descriptively without inferential testing. Absence of a statistically significant difference does not establish equivalence. ICU, intensive care unit; IgE, immunoglobulin E; WBC, white blood cell count.

**Figure 1:**
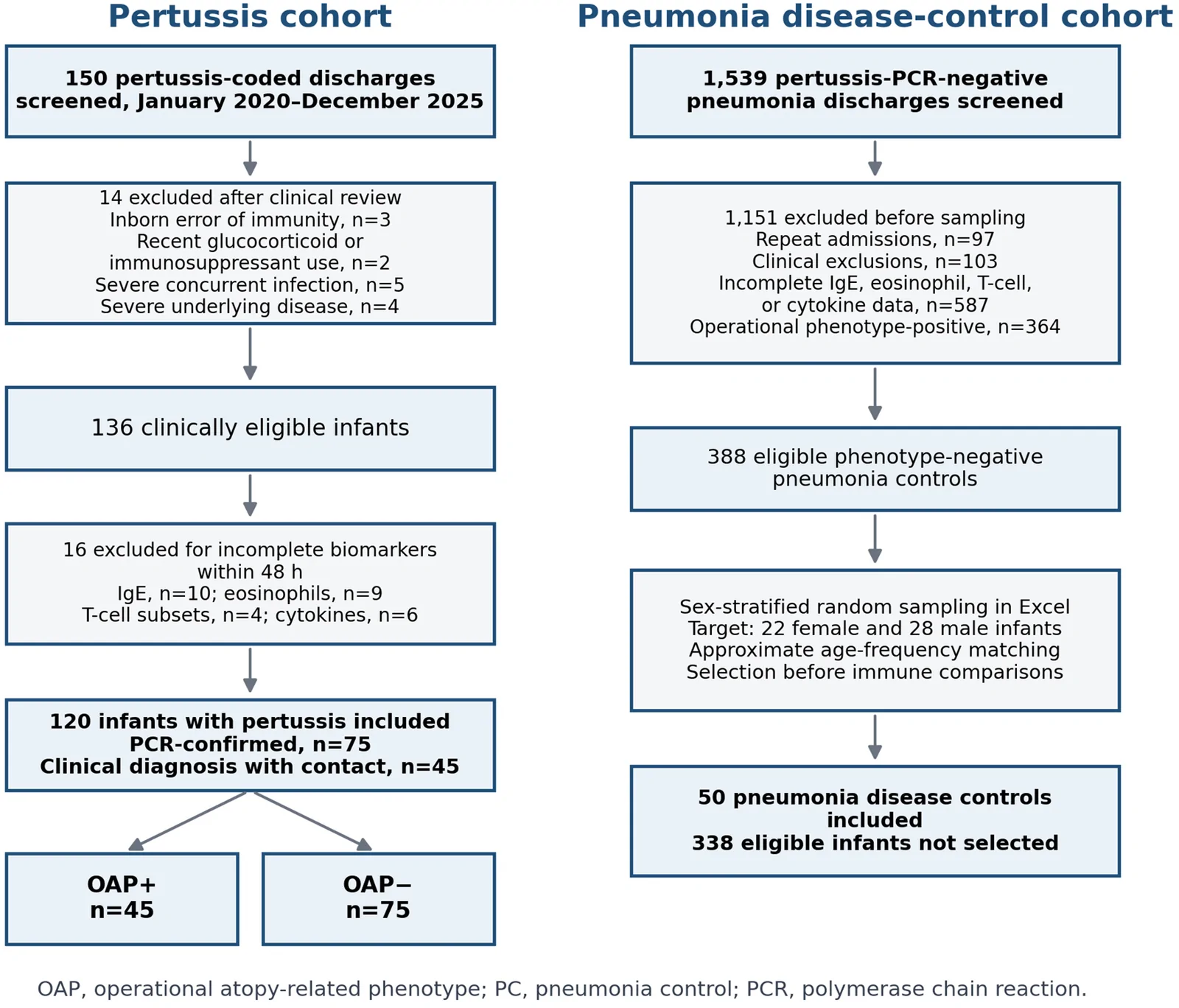
Study flow diagram. OAP, operational atopy-related phenotype; PC, pneumonia control; PCR, polymerase chain reaction.

### 3.2 Phenotype components

All 45 OAP + infants had IgE >15 IU/mL; 36 (80.0%) had eosinophilia, 32 (71.1%) had eczema, and 23 (51.1%) had parental allergic history. Eight infants had exactly one qualifying feature in addition to elevated IgE, 28 had exactly two, and 9 had all three. The recorded group labels exactly matched the prespecified composite when recalculated from the source variables. Full overlap patterns are shown in Supplementary Table S4.

### 3.3 Three-group immune comparisons

The OAP + group had higher unadjusted CD4 + percentages and CD4/CD8 ratios than both comparison groups. IFN-*γ* was lower in OAP + than OAP− infants, IL-6 was higher in OAP + than both comparison groups, and the IFN-*γ*/IL-6 ratio was lowest in OAP + infants (Tables 2, 3). The pneumonia group provided clinical context only; the study design did not test whether these contrasts were pertussis-specific.

**Table 2:** Peripheral blood lymphocyte subsets in the three study groups.

Measure	OAP + (*n* = 45)	OAP− (*n* = 75)	PC (*n* = 50)	Statistic	*P* value
CD3+ T cells, %	68.1 ± 6.7	66.2 ± 9.2	66.7 ± 7.1	0.766	0.467
CD4+ T cells, %	45.0 ± 6.2ᵃ,ᵇ	39.9 ± 5.7	40.2 ± 6.1	11.705	<0.001
CD8+ T cells, %	21.9 ± 4.8	24.0 ± 5.5	24.0 ± 4.2	3.007	0.052
CD4/CD8 ratio	2.18 ± 0.66ᵃ,ᵇ	1.76 ± 0.57	1.74 ± 0.45	9.262	<0.001
CD19+ B cells, %	16.5 ± 3.5	15.3 ± 3.8	17.0 ± 4.0	3.388	0.036
NK cells, %	13.0 ± 4.6	14.1 ± 4.7	14.0 ± 4.4	0.986	0.375

Values are mean ± standard deviation. Overall *P* values are from one-way analysis of variance; the statistic column reports F. Superscripts denote Bonferroni-adjusted pairwise *P* < 0.05: ^ᵃ^ vs. OAP−; ^ᵇ^ vs. PC; ^ᶜ^ OAP− versus PC.

**Table 3:** Serum cytokines in the three study groups.

Measure	OAP + (*n* = 45)	OAP− (*n* = 75)	PC (*n* = 50)	Statistic	*P* value
IFN-*γ*, pg/mL	10.88 (7.14–14.71)ᵃ	14.69 (9.77–19.41)ᶜ	11.09 (6.49–14.88)	14.166	<0.001
IL-6, pg/mL	32.67 (24.66–46.21)ᵃ,ᵇ	14.24 (11.38–20.62)	17.01 (12.26–23.82)	38.083	<0.001
TNF-α, pg/mL	4.66 (3.12–7.81)	5.50 (3.83–8.99)	5.53 (3.27–8.50)	0.405	0.817
IL-2, pg/mL	2.32 (1.34–4.63)	3.42 (2.03–6.31)	2.48 (1.54–3.73)	6.959	0.031
IFN-γ/IL-6 ratio	0.327 (0.201–0.523)ᵃ,ᵇ	0.908 (0.576–1.652)ᶜ	0.639 (0.270–1.272)	38.316	<0.001

Values are median (interquartile range). Overall *P* values are from Kruskal–Wallis tests; the statistic column reports H. Superscripts denote Bonferroni-adjusted pairwise *P* < 0.05: ^ᵃ^ versus OAP−; ^ᵇ^ versus PC; ^ᶜ^ OAP− versus PC.

### 3.4 Adjusted primary immune comparisons

After adjustment for chronological age, sex, PCR status, cough duration before sampling, and admission year, OAP + status was associated with a higher CD4 + percentage, lower CD8 + percentage, and higher CD4/CD8 ratio (all q < 0.05). OAP + status was also associated with lower IFN-*γ*, higher IL-6, lower IL-2, and a lower IFN-*γ*/IL-6 ratio (all q < 0.05). CD3+, CD19+, NK-cell percentage, and TNF-α did not meet the corrected significance criterion (Table 4). Covariate variance inflation factors ranged from 1.01 to 1.05.

**Table 4:** Adjusted associations of the operational phenotype with immune outcomes in the pertussis cohort.

Family	Outcome	Effect	Estimate (95% CI)	*P* value	BH q value
Lymphocyte	CD3+ T cells, %	Adjusted mean difference	2.11 (−0.84–5.07)	0.160	0.169
Lymphocyte	CD4+ T cells, %	Adjusted mean difference	5.25 (2.92–7.57)	<0.001	<0.001
Lymphocyte	CD8+ T cells, %	Adjusted mean difference	−2.36 (−4.38–−0.34)	0.022	0.045
Lymphocyte	CD4/CD8 ratio	Adjusted mean difference	0.441 (0.205–0.677)	<0.001	<0.001
Lymphocyte	CD19+ B cells, %	Adjusted mean difference	1.15 (−0.34–2.64)	0.130	0.169
Lymphocyte	NK cells, %	Adjusted mean difference	−1.22 (−2.96–0.52)	0.169	0.169
Cytokine	IFN-γ, pg/mL	Adjusted geometric mean ratio	0.72 (0.58–0.90)	0.004	0.006
Cytokine	IL-6, pg/mL	Adjusted geometric mean ratio	2.07 (1.65–2.59)	<0.001	<0.001
Cytokine	TNF-α, pg/mL	Adjusted geometric mean ratio	0.93 (0.72–1.21)	0.610	0.610
Cytokine	IL-2, pg/mL	Adjusted geometric mean ratio	0.70 (0.52–0.94)	0.016	0.020
Cytokine	IFN-γ/IL-6 ratio	Adjusted geometric mean ratio	0.348 (0.252–0.482)	<0.001	<0.001

Reference group: OAP−. Models included chronological age, sex, PCR status, cough duration before sampling, and admission year. Lymphocyte outcomes were modeled on their original scale with heteroscedasticity-consistent (HC3) standard errors; cytokines were log-transformed and exponentiated. Benjamini–Hochberg adjustment was applied separately to the six lymphocyte outcomes and five cytokine outcomes. CI, confidence interval; NK, natural killer; BH, Benjamini–Hochberg.

### 3.5 Sensitivity and selection analyses

A stricter definition requiring elevated IgE plus at least two additional phenotype components classified 37 infants as positive and retained 75 original OAP− comparators; eight intermediate cases were excluded. Associations remained in the same direction for CD4 + percentage (adjusted mean difference 5.82, 95% CI 3.26–8.39), CD4/CD8 ratio (0.42, 0.19–0.64), IFN-*γ* (geometric mean ratio 0.74, 0.59–0.93), IL-6 (2.23, 1.76–2.82), and the IFN-*γ*/IL-6 ratio (0.33, 0.24–0.46), all q < 0.05. Component-based definitions showed the same core directions (Supplementary Table S6).

Among the 75 PCR-confirmed cases, OAP + status remained associated with CD4 + percentage (5.31, 95% CI 1.87–8.75), CD4/CD8 ratio (0.41, 0.16–0.66), IFN-*γ* (0.64, 0.50–0.82), IL-6 (1.93, 1.39–2.67), and the ratio (0.33, 0.21–0.52), all q < 0.01. Included cases and the 16 biomarker-incomplete infants showed no statistically significant differences in observed age, sex, PCR status, epidemiological contact, length of stay, cough duration, peak WBC, or specialty-ICU care; this does not exclude selection bias from unmeasured factors (Supplementary Table S5).

### 3.6 Exploratory correlations and length of stay

In the complete pertussis cohort, the IFN-*γ*/IL-6 ratio was inversely correlated with length of stay (Spearman r = −0.233, *P* = 0.010). The estimate remained negative in OAP + infants but was not statistically significant (r = −0.272, *P* = 0.071), whereas estimates were positive and non-significant in OAP− infants (r = 0.099, *P* = 0.397) and pneumonia controls (r = 0.022, *P* = 0.880). Significant pooled correlations of IgE with IL-6 and the ratio were absent within the pertussis subgroups, consistent with between-group separation rather than an individual-level dose-response relationship (Supplementary Table S7).

Hospital days ranged from 5 to 29 (median 11; mean 12.51; variance 24.55). In the combined negative-binomial model, OAP + status was associated with longer hospitalization (adjusted IRR 1.33, 95% CI 1.14–1.55; *P* < 0.001), whereas the IFN-*γ*/IL-6 ratio was not independently associated (IRR 0.98 per doubling, 95% CI 0.92–1.03; *P* = 0.403) (Table 5). A gamma model yielded a similar phenotype mean ratio of 1.34 (95% CI 1.14–1.57), while the median-dichotomized sensitivity analysis was less precise and not statistically significant. These analyses were exploratory and did not include unavailable severity and treatment covariates.

**Table 5:** Exploratory negative-binomial regression of hospital length of stay among 120 infants with pertussis.

Variable	Adjusted IRR (95% CI)	*P* value
Male sex	1.072 (0.947–1.214)	0.270
OAP + phenotype	1.328 (1.137–1.550)	<0.001
IFN-γ/IL-6 ratio, per doubling	0.976 (0.921–1.034)	0.403
Age, per day	0.999 (0.995–1.003)	0.565
PCR-confirmed pertussis	0.965 (0.847–1.100)	0.595
Cough duration before sampling, per day	0.996 (0.990–1.001)	0.114
Admission year, per calendar year	1.012 (0.976–1.049)	0.510

The continuous count outcome was total hospital days, including days in a specialty ICU. The model included all variables shown. A log2 transformation was used for the IFN-γ/IL-6 ratio, so its IRR represents a doubling of the ratio. The Poisson Pearson dispersion was 1.58 and the estimated negative-binomial dispersion parameter was 0.0366 (*P* = 0.001). CI, confidence interval; IRR, incidence rate ratio.

## 4 Discussion

### 4.1 Principal findings

A prespecified operational atopy-related phenotype identified differences in selected peripheral immune measurements among hospitalized infants aged 1–3 months with pertussis. Associations with higher CD4 + percentage and CD4/CD8 ratio, lower IFN-*γ*, higher IL-6, and a lower IFN-*γ*/IL-6 ratio persisted after adjustment, false-discovery-rate correction, a stricter phenotype definition, and restriction to PCR-confirmed cases. OAP + status was also associated with longer hospitalization in an exploratory count model. These results support immune heterogeneity associated with the local phenotype, but they do not validate an atopy diagnosis, demonstrate a pertussis-specific interaction, or establish a prognostic test.

### 4.2 Biological interpretation

Natural pertussis infection in very young infants can induce IFN-*γ*-dominant type 1 responses (9), whereas early pediatric atopic dermatitis can show type 2-centered inflammation with type 17 and type 22 activity and limited type 1 upregulation (10). The lower IFN-*γ* and higher IL-6 in OAP + infants are compatible with host-related immune variation. IL-6 is pleiotropic and contributes to several inflammatory pathways, including conditions that favor type 17 differentiation (13); it is not a lineage-specific type 2 marker. *Bordetella pertussis* products can also induce IL-6 and alter T-cell polarization (14). Because IL-4, IL-5, IL-13, IL-17A, IL-22, antigen-specific responses, and functional assays were not measured, the observed pattern cannot be labeled direct evidence of Th2 or Th17 polarization.

IL-6 was higher in OAP + pertussis than in pneumonia controls, but the control group was clinically heterogeneous: 22% were classified as viral, 38% bacterial, 26% mixed, and 14% undetermined. IL-6 also varies with infection type, severity, and sampling time. Moreover, no phenotype-positive pneumonia group was included. The control comparison therefore contextualizes the distributions but cannot show that the phenotype effect is specific to pertussis rather than a broader host response across respiratory infections.

### 4.3 Phenotype validity and case ascertainment

The composite deliberately required both elevated total IgE and at least one independent clinical or laboratory feature. The overlap and sensitivity analyses reduce concern that a single component entirely drove the findings, but the thresholds were local age-specific reference limits rather than validated diagnostic criteria. Acute infection can influence eosinophils and immune mediators, and recorded eczema or parental history may be incomplete. OAP− should therefore be read as operational phenotype-negative, not non-atopic.

The cohort combined 75 PCR-confirmed cases with 45 PCR-negative clinical cases who all had epidemiological contact. Results were retained in PCR-confirmed cases, which reduces but does not eliminate ascertainment concerns. Pharyngeal swabs were used, while nasopharyngeal sampling is generally preferred for pertussis PCR (12); some PCR-negative infants may therefore have been misclassified by laboratory testing. Qualitative results also prevented analysis by organism burden.

### 4.4 Length of stay and clinical implications

Analyzing hospital days continuously avoided the information loss of a median split. The negative-binomial model indicated approximately 33% longer expected stay for OAP + infants after adjustment for available covariates, but the cytokine ratio was not independently associated. The phenotype-only and ratio-only models each showed associations, illustrating how correlated explanatory signals can change when modeled together. Length of stay depends on disease severity, treatment, respiratory support, discharge practice, social factors, and calendar period. Because several of these factors were unavailable, the IRR should be interpreted as an exploratory adjusted association rather than an independent causal effect or prediction model.

The current clinical contribution is interpretive: host atopy-related features may contribute to variation in lymphocyte percentages and cytokine concentrations during infant pertussis. The results do not support changing treatment, extending hospitalization, or using the IFN-*γ*/IL-6 ratio as a bedside threshold. Prospective studies should collect vaccination, prematurity, pretreatment, respiratory-support, standardized severity, and longitudinal allergy outcomes before clinical application is considered.

### 4.5 Strengths and limitations

Strengths include a narrow high-risk age range, immune sampling within 48 h, explicit PCR and clinical case counts, retention of infants who later required specialty-ICU care, transparent two-stage control selection, prespecified primary outcome families, adjusted effect estimates with confidence intervals, multiplicity control, and several sensitivity analyses.

The study also has substantial limitations. Its retrospective single-center complete-case design creates selection and information bias. Although the 16 biomarker-incomplete infants did not differ detectably on available variables, reasons for non-completion were not recorded and unmeasured differences remain possible. The operational phenotype is unvalidated, uses local reference limits, and was assessed during acute illness. The control group excluded phenotype-positive pneumonia and healthy infants, and its infection categories were based on nonuniform clinically directed testing and physician judgment. The disease-control comparison therefore cannot support a pertussis-specific mechanism.

Prematurity, gestational age, individual infant and maternal vaccination, pre-admission macrolide exposure, detailed respiratory support, standardized severity, and complete coinfection testing were unavailable, leaving residual confounding. Severe concurrent infections were excluded, but unrecognized coinfection may remain. Pharyngeal rather than nasopharyngeal swabs and qualitative PCR could reduce laboratory sensitivity. Immune measurements were limited to percentages, total IgE, eosinophils, and four cytokines; absolute lymphocyte counts, antigen-specific antibodies, and lineage-defining cytokines were not assessed. Finally, no formal power calculation was performed, exploratory correlations were not multiplicity-adjusted, and external validity is limited to similar hospitalized infants.

## 5 Conclusions

Among infants aged 1–3 months hospitalized with pertussis, a prespecified operational atopy-related phenotype was associated with selected peripheral T-cell and cytokine differences after adjustment for available confounders. The phenotype was also associated with longer hospital stay in an exploratory count model, whereas the continuous IFN-*γ*/IL-6 ratio was not independently associated. These findings are hypothesis-generating and require prospective validation with standardized phenotype assessment, broader immune measurements, quantitative pathogen testing, and complete clinical severity data.

## Data Availability

The individual-level datasets are not publicly available because they contain sensitive pediatric health information and are subject to institutional ethics restrictions. De-identified data may be made available by the corresponding author upon reasonable request and with approval from the institutional ethics committee. Requests should be directed to Lingyun Zhang, 444072587@qq.com.
