# Supplementary material for: Peripheral immune profiles associated with an operational atopy-related phenotype in young infants with pertussis: a single-center retrospective study
Source: Front Pediatr. 2026 Oct 29;14:1987917. doi: 10.3389/fped.2026.1987917 (PMC13754880; doi:10.3389/fped.2026.1987917)
Supplement: Supplementary file 1 [file Supplementaryfile1.pdf]

Supplementary material

Peripheral immune profiles associated with an operational atopy-related phenotype in young infants with pertussis: a single-center retrospective study

Supplementary Table S1. Detailed participant screening and allocation.

Cohort	Stage	Number	Disposition
Pertussis	Pertussis-coded discharges screened	150	Assessed for eligibility
Pertussis	Inborn error of immunity	3	Excluded
Pertussis	Recent glucocorticoid/immunosuppressant use	2	Excluded
Pertussis	Severe concurrent infection	5	Excluded
Pertussis	Severe underlying disease	4	Excluded
Pertussis	Incomplete biomarkers within 48 h	16	Excluded
Pertussis	Included	120	OAP+, n=45; OAP−, n=75
Pneumonia	Pertussis-PCR-negative candidates	1,539	Screened
Pneumonia	Repeat admissions	97	Excluded
Pneumonia	Clinical exclusions	103	Excluded
Pneumonia	Incomplete IgE/eosinophil/T-cell/cytokine data	587	Excluded
Pneumonia	Operational phenotype-positive	364	Excluded
Pneumonia	Eligible before sampling	388	Sampling frame
Pneumonia	Sex-stratified random sample with approximate age-frequency matching	50	Included; 338 not selected

Supplementary Table S2. Operational definitions and coding.

Variable	Definition
Chronological age	Actual postnatal age in days; corrected age was not used.
Pertussis case	Age 1–3 months plus positive IS481 PCR from an admission-day pharyngeal swab, or PCR-negative clinical diagnosis under the 2024 Chinese criteria with documented epidemiological contact.
OAP+	Total IgE >15 IU/mL plus at least one of eosinophils >0.4×10^9/L, documented eczema, or allergic disease in either parent.
OAP−	Eligible pertussis case not meeting the full composite; not synonymous with non-atopic.
Cough duration	Days of cough before admission or immune sampling.
Hospital days	Total admission-to-discharge days, including specialty-ICU days.
Pneumonia category	Treating physician's final clinical judgment integrating symptoms, CRP/blood count, imaging, and available microbiology: viral, bacterial, mixed, or unidentified/undetermined.
Complete case	All variables required for the reported analysis were observed; no imputation.

Supplementary Table S3. Pertussis ascertainment by operational phenotype.

Ascertainment	OAP+ (n=45)	OAP− (n=75)	Total (n=120)	P value
PCR-confirmed	26 (57.8)	49 (65.3)	75 (62.5)	0.440
PCR-negative clinical diagnosis with contact	19 (42.2)	26 (34.7)	45 (37.5)	—

Values are n (%). P value is from Fisher exact test for the 2×2 ascertainment table.

Supplementary Table S4. Components and overlap among 45 OAP+ infants.

Pattern	n (%)
IgE >15 IU/mL	45 (100.0)
Eosinophilia	36 (80.0)
Eczema	32 (71.1)
Parental allergic history	23 (51.1)
Exactly one additional feature	8 (17.8)
Eosinophilia only	5 (11.1)
Eczema only	2 (4.4)
Parental history only	1 (2.2)
Exactly two additional features	28 (62.2)
Eosinophilia + eczema only	15 (33.3)
Eosinophilia + parental history only	7 (15.6)
Eczema + parental history only	6 (13.3)
All three additional features	9 (20.0)

All infants in this table also had total IgE >15 IU/mL.

Supplementary Table S5. Available characteristics of included and biomarker-incomplete pertussis infants.

Characteristic	Included (n=120)	Excluded (n=16)	P value
Age, days	59.5 (48.0–72.0)	63.0 (45.5–72.5)	0.968
Length of stay, days	11.0 (9.0–15.0)	10.5 (9.0–13.2)	0.616
Cough duration before sampling, days	19.0 (15.0–24.0)	20.0 (16.0–28.5)	0.431
Peak WBC, $\times 10^9$/L	27.0 (17.2–36.6)	26.0 (21.2–30.1)	0.736
Male sex	67 (55.8)	10 (62.5)	0.789
PCR-positive	75 (62.5)	9 (56.2)	0.785
Epidemiological contact	100 (83.3)	12 (75.0)	0.483
Specialty-ICU care	11 (9.2)	2 (12.5)	0.651

Continuous values are median (interquartile range) and were compared by Mann–Whitney U tests; categorical values are n (%) and were compared by Fisher exact tests. Non-significant tests do not demonstrate equivalence. Among the 16 excluded infants, IgE was missing in 10, eosinophils in 9, T-cell subsets in 4, and cytokines in 6; domains could overlap.

Supplementary Table S6A. Core sensitivity analyses.

Outcome	Strict phenotype estimate (95% CI)	q	PCR-confirmed estimate (95% CI)	q
CD4+ percentage	5.822 (3.259–8.386)	<0.001	5.309 (1.867–8.752)	0.008
CD4/CD8 ratio	0.417 (0.193–0.641)	<0.001	0.409 (0.156–0.663)	0.008
IFN-γ	0.735 (0.585–0.925)	0.014	0.640 (0.502–0.816)	<0.001
IL-6	2.228 (1.764–2.815)	<0.001	1.928 (1.390–2.674)	<0.001
IFN-γ/IL-6 ratio	0.330 (0.237–0.459)	<0.001	0.332 (0.213–0.517)	<0.001

Strict phenotype: IgE >15 IU/mL plus at least two additional components (37 positive, 75 original OAP− comparators; eight intermediate cases excluded). PCR-confirmed analysis: 26 OAP+ and 49 OAP−. Estimates are adjusted mean differences for CD4+ percentage and CD4/CD8 ratio, and adjusted geometric mean ratios for cytokines. Models used the covariates available within each sensitivity sample.

Supplementary Table S6B. Core immune outcomes under component-based phenotype definitions.

Component definition	Outcome	Adjusted estimate (95% CI)	P	BH q
IgE + eosinophilia (36 vs 75)	CD4+ percentage	5.727 (3.070–8.383)	<0.001	<0.001
IgE + eosinophilia (36 vs 75)	CD4/CD8 ratio	0.488 (0.221–0.756)	<0.001	0.001
IgE + eosinophilia (36 vs 75)	IFN-γ	0.693 (0.541–0.887)	0.004	0.006
IgE + eosinophilia (36 vs 75)	IL-6	2.249 (1.757–2.879)	<0.001	<0.001
IgE + eosinophilia (36 vs 75)	IFN-γ/IL-6 ratio	0.308 (0.215–0.441)	<0.001	<0.001
IgE + eczema (32 vs 75)	CD4+ percentage	5.120 (2.507–7.733)	<0.001	<0.001
IgE + eczema (32 vs 75)	CD4/CD8 ratio	0.356 (0.112–0.600)	0.004	0.013
IgE + eczema (32 vs 75)	IFN-γ	0.752 (0.580–0.973)	0.030	0.038
IgE + eczema (32 vs 75)	IL-6	2.282 (1.775–2.934)	<0.001	<0.001
IgE + eczema (32 vs 75)	IFN-γ/IL-6 ratio	0.329 (0.231–0.469)	<0.001	<0.001
IgE + parental history (23 vs 75)	CD4+ percentage	4.858 (2.083–7.632)	<0.001	0.004
IgE + parental history (23 vs 75)	CD4/CD8 ratio	0.373 (0.109–0.636)	0.006	0.017
IgE + parental history (23 vs 75)	IFN-γ	0.768 (0.609–0.969)	0.026	0.044
IgE + parental history (23 vs 75)	IL-6	2.012 (1.562–2.592)	<0.001	<0.001
IgE + parental history (23 vs 75)	IFN-γ/IL-6 ratio	0.382 (0.264–0.552)	<0.001	<0.001

Each definition requires total IgE >15 IU/mL plus the named component. Original OAP− infants (n=75) are the comparator for all three analyses; OAP+ infants not meeting the component definition are excluded rather than reclassified as negative. Estimates are adjusted mean differences for CD4+ percentage (percentage points) and CD4/CD8 ratio, and adjusted geometric mean ratios for cytokines. Models include age, sex, PCR status, cough duration before sampling, and admission year, with HC3 robust standard errors. BH q values are calculated within the full six-outcome lymphocyte and five-outcome cytokine families for each definition; the table shows the five core outcomes discussed in the article.

Supplementary Table S7. Exploratory Spearman correlations.

Variable pair	Pertussis cohort	OAP+	OAP−	PC
IFN-γ/IL-6 ratio vs length of stay	-0.233 (0.010)	-0.272 (0.071)	0.099 (0.397)	0.022 (0.880)
IL-6 vs pre-sampling cough duration	0.259 (0.004)	0.352 (0.018)	0.293 (0.011)	-0.181 (0.208)
Total IgE vs IFN-γ/IL-6 ratio	-0.496 (<0.001)	0.003 (0.985)	-0.114 (0.329)	-0.170 (0.239)
Total IgE vs IL-6	0.452 (<0.001)	-0.209 (0.168)	0.109 (0.351)	0.313 (0.027)

Values are Spearman r (P value). No multiplicity correction was applied. Pooled IgE correlations reflect both within-group and between-group variation and should not be interpreted as within-group dose-response effects.

Supplementary Table S8. Length-of-stay model diagnostics and sensitivity analyses.

Analysis	Phenotype estimate (95% CI)	P value	Ratio estimate (95% CI)	P value
Negative-binomial main model	1.328 (1.137–1.550)	<0.001	0.976 (0.921–1.034)	0.403
Gamma log-link sensitivity	1.338 (1.144–1.566)	<0.001	0.981 (0.929–1.037)	0.495
Median-dichotomized logistic sensitivity	2.288 (0.898–5.832)	0.083	1.615 (0.652–3.998)	0.300

Negative-binomial and gamma models used continuous hospital days and the cytokine ratio per doubling. The logistic sensitivity defined prolonged stay as >11 days and low ratio as <0.635. All models adjusted for chronological age, sex, PCR status, cough duration before sampling, and admission year.

Supplementary Table S9. Clinical etiologic classification of 50 pneumonia controls.

Category	n (%)
Viral	11 (22.0)
Bacterial	19 (38.0)
Mixed	13 (26.0)
Unidentified/undetermined	7 (14.0)

Categories were based on the treating physician's final judgment integrating symptoms, CRP/blood count, imaging, and available pathogen testing. Testing was clinically directed rather than uniform; categories are descriptive only.

Supplementary notes

Specialty-ICU handling. Eleven included infants entered a specialty ICU approximately 2–3 days after admission and after immune sampling because of pertussis progression, respiratory failure, or declining oxygen saturation. They remained in the cohort, and their total hospital days included specialty-ICU care. The five severe-concurrent-infection exclusions were distinct infants who required immediate general-ICU transfer before baseline immune testing.

Control sampling. Excel random numbers were generated within sex strata after the target of 22 female and 28 male controls had been set; age-frequency matching was approximate. The immune comparison results were not viewed before selection. This was not propensity-score matching or individual matching.

Data completeness. Analyses used complete records without imputation. Reasons for non-completion of immune testing among the 16 pertussis exclusions were not systematically recorded.
